# Influence of the Microstructure and Optical Constants on Plasmonic Properties of Copper Nanolayers

**DOI:** 10.3390/ma14237292

**Published:** 2021-11-29

**Authors:** Tomasz Rerek, Beata Derkowska-Zielinska, Marek Trzcinski, Robert Szczesny, Mieczyslaw K. Naparty, Lukasz Skowronski

**Affiliations:** 1 Institute of Mathematics and Physics, Bydgoszcz University of Science and Technology, Kaliskiego 7, 85-796 Bydgoszcz, Poland; marek.trzcinski@pbs.edu.pl (M.T.); mieczyslaw.naparty@pbs.edu.pl (M.K.N.); lukasz.skowronski@pbs.edu.pl (L.S.); 2 Institute of Physics, Faculty of Physics, Astronomy and Informatics, Nicolaus Copernicus University in Torun, Grudziadzka 5, 87-100 Torun, Poland; beata@fizyka.umk.pl; 3 Faculty of Chemistry, Nicolaus Copernicus University in Torun, Gagarina 7, 87-100 Torun, Poland; robert.szczesny@umk.pl

**Keywords:** thin copper layers, optical properties, microstructure

## Abstract

Copper layers with thicknesses of 12, 25, and 35 nm were thermally evaporated on silicon substrates (Si${}_{\left(100\right)}$) with two different deposition rates 0.5 and 5.0 Å/s. The microstructure of produced coatings was studied using atomic force microscopy (AFM) and powder X-ray diffractometer (XRD). Ellipsometric measurements were used to determine the effective dielectric functions <$\tilde{\varepsilon }$> as well as the quality indicators of the localized surface plasmon (LSP) and the surface plasmon polariton (SPP). The composition and purity of the produced films were analysed using X-ray photoelectron spectroscopy (XPS).

## 1. Introduction

Copper is commonly used in electronics due to its high electrical conductivity and low cost [1]. The optical properties of noble metals and their nanostructures have led to the development of materials for, e.g., optical nanosensors and in surface-enhanced spectroscopies [1,2,3,4,5]. In these systems, an optical response in the form of a localized surface plasmon (LSP) is used. The occurrence of LSP is theoretically possible for all metals, semiconductors, and their alloys, which have a large negative real dielectric constant and a small imaginary dielectric constant. However, gold and silver are the most commonly used in plasmonics [6], whereas the low cost of copper (compared to Au and Ag) means that Cu is currently gaining popularity in plasmonic applications [7,8]. However, the main disadvantage of this material is the formation of an oxide layer [9], which, in the case of plasmonic applications, is not desirable, because such a layer strongly suppresses the localized surface plasmon (LSP) [6]. The Q${}_{LSP}$ quality factor is a measure of the quality of LSP. McPeak et al. obtained, for copper deposited at rate of 35 Å/s at pressure of 3 × 10${}^{-8}$ Tor, a value of Q${}_{LSP}$ of about 44 and 40 for a wavelength range of 650 nm and 1000 nm, respectively, [10].

The microstructure and, consequently, the optical properties (including plasmonic) of the produced metallic layers are influenced by many factors such as the properties of the deposited material and the used substrate (e.g., reactivity, surface energies, wettability). It is also important to choose the method and parameters of the layer deposition (deposition rate, atmosphere (or vacuum level—residual gases [11]), substrate temperature, etc.) [10]. The selection of appropriate PVD (physical vapor deposition) conditions and parameters is crucial to optimize plasmonic film properties. As shown by McPeak [10], with proper optimization of the deposition rate, the quality factor $Q_{LSP}$ on copper layers can have similar values or even higher (for wavelengths above 1000 nm) than a gold layer of the same thickness. The authors also indicated that the better performance than reported by Palik (for Au, Ag, and Cu) [12] and Rakić (for Al) [13] may be due to the fact that their layers are much thicker, which makes the grains much larger, and this may result in lower losses at the grain boundaries scattering.

In this research, we investigated the optical and plasmonic properties of thermally deposited copper nanolayers obtained with low rates at low vacuum conditions. Then, the microstructure of the obtained coatings was examined using the atomic force microscopy (AFM) and powder X-ray diffractometry (XRD). We have shown how the dielectric functions as well as the Q${}_{LSP}$ (quality factor of localized surface plasmon) and Q${}_{SPP}$ (quality factor of surface plasmon polariton) of Cu films change with thickness and deposition rate. These properties were determined on the basis of ellipsometric measurements. Low-vacuum formation of layers is associated with a risk of residual gas contamination, so we performed X-ray photoelectron spectroscopy (XPS) studies to investigate the concentration of oxygen in the produced films.

## 2. Materials and Methods

The copper thin layers were evaporated on the polished silicon wafers (Si${}_{100}$) using the thermal evaporation technique, where the pressure was below 2 × 10${}^{-5}$ mbar [14,15,16]. Pieces of Cu (99.99%) wire were placed in a molybdenum spiral evaporator. Samples ware deposited at two deposition rates: 0.5 and 5.0 Å/s. The thicknesses of the copper films were 12, 25, and 35 nm. The deposition rate and thickness were controlled by a QCM—quartz crystal microbalance (6.0 MHz), which, together with the substrate holder, was 20 cm above the spiral evaporator.

The surface topography of the obtained coatings was examined using an atomic force microscope (AFM) Innova from Bruker (Billerica, MA, USA) with standard silicon tips dedicated for the tapping mode. The surface roughness parameters were calculated based on the AFM images of 1 μm × 1 μm area using the NanoScope Analysis software (version 1.40). The average roughness ($R_{a}$) and the root mean square roughness ($R_{q}$) were defined as:

(1) $$R_{a}=\frac{1}{N}\sum_{k=1}^{N}\left|Z_{k}\right|,$$

and

(2) $$R_{q}=\sqrt{\frac{\sum_{k=1}^{N}{\left|Z_{k}\right|}^{2}}{N}},$$

where Z${}_{k}$—the current surface height value, and *N*—the number of measured points.

The powder X-ray diffraction (XRD) patterns were performed using the Phillips X’Pert (Malvern Panalytical Ltd., Malvern, UK) system with Cu K${}_{\alpha }$ radiation (wavelength 1.5418 Å) and the X’Celerator Scientific (Malvern Panalytical Ltd., Malvern, UK) detector. These measurements were made in the range from 2θ = 15 to 90${}^{\circ }$.

The chemical composition of the deposited layers was estimated using the X-ray photoelectron spectroscopy (XPS) technique. The photoelectrons were detected by a VG-Scienta R3000 (Uppsala, Sweden) spectrometer with energy step set at ΔE = 0.2 eV. The XPS measurements were performed in an ultra-high vacuum (UHV, base pressure below 2 × 10${}^{-10}$ mbar) using an Al K${}_{\alpha }$ source (1486.6 eV). The dwelling time was 100 ms, and the number of scans was 15 iterations for each region. Spectra were analyzed using CasaXPS software (v. 2.3.16, Casa Software Ltd., Teignmouth, UK).

The thickness, optical constants, and plasmonic properties of the prepared Cu thin films were investigated by means of the V-VASE device from J.A. Woollam Co., Inc. (Lincoln, NE, USA). Ellipsometric azimuths (Ψ, Δ) were measured for three angles of incidence light (65${}^{\circ }$, 70${}^{\circ }$, and 75${}^{\circ }$) in the spectral range 193–2000 nm (0.6–6.5 eV). The analysis of ellipsometric data was performed using the WVASE32 software. The complex dielectric function fully describes electronic response of a material and is given by a formula:

(3) $$\begin{array}{c} \tilde{\varepsilon }=\varepsilon_{1}+i\varepsilon_{2}, \end{array}$$

where $\varepsilon_{1}$ and $\varepsilon_{2}$ are real and imaginary parts of the dielectric function, respectively. If, for a certain frequency, the real part of the dielectric function ($\varepsilon_{1}$) is less than zero, it is possible to excite the surface plasmon resonance, the quality of which will depend on the imaginary part of the dielectric function ($\varepsilon_{2}$). On the basis of these two parts of the dielectric function, it is possible to determine the quality factors for the localized surface plasmon (Q${}_{LSP}$) and the surface plasmon polaritons (Q${}_{SPP}$). The quality factors are described by the following formulas [17]:

(4) $$\begin{array}{c} Q_{LSP}=-\varepsilon_{1}/\varepsilon_{2}, \end{array}$$

and

(5) $$\begin{array}{c} Q_{SPP}=\varepsilon_{1}^{2}/\varepsilon_{2}. \end{array}$$

## 3. Results and Discussion

**Figure 1:**
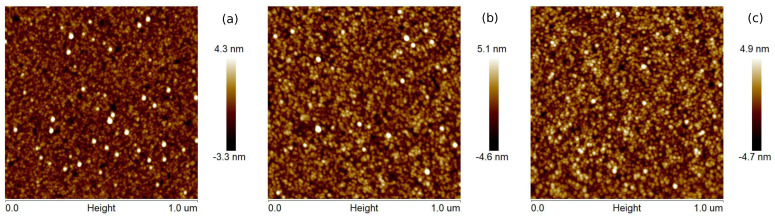
AFM images (1 μm × 1 μm) of the (**a**) 12 nm, (**b**) 25 nm, and (**c**) 35 nm Cu films prepared at the deposition rate 0.5 Å/s.

**Figure 2:**
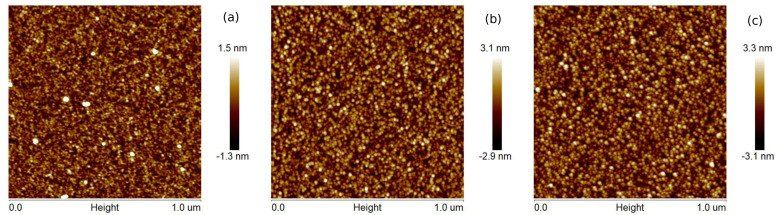
AFM images (1 μm × 1 μm) of the (**a**) 12 nm, (**b**) 25 nm, and (**c**) 35 nm Cu films prepared at the deposition rate 5.0 Å/s.

The surface topographies of Cu layers with thicknesses of 12, 25, and 35 nm produced at evaporation rates of 0.5 and 5.0 Å/s are presented in Figure 1 and Figure 2, respectively. The films produced with a lower evaporation rate are made of grains of larger lateral sizes than that the layers fabricated with the deposition rate of 5 Å/s. Generally, the largest size of grains is about 2 and 4 nm for *v* = 0.5 and 5 Å/s, respectively. For the coatings obtained at the rate of 0.5 Å/s, the roughness parameters—the average roughness ($R_{q}$) and the root mean square roughness ($R_{a}$)—are even twice as high as for the layers obtained at the deposition rate of 5 Å/s (see Table 1). These parameters also increase with increasing thickness of the copper films. Generally, the obtained layers are relatively smooth, and the roughness parameter values are below 1.4 nm. The maximum roughness ($R_{max}$) of Cu coatings is in the range 12.7 to 14.4 nm and 6.1 to 7.6 nm for *v* values of 0.5 and 5 Å/s, respectively.

**Figure 3:**
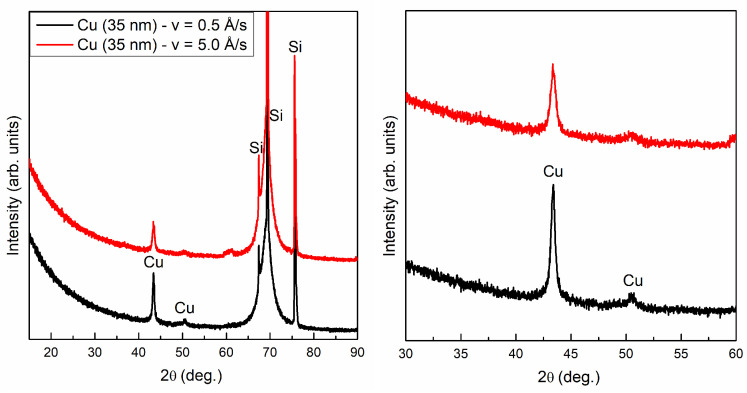
XRD patterns for 35 nm Cu films deposited with rate 0.5 and 5.0 Å/s. All peaks marked with an ellipse orginate from the silicon substrate.

The XRD patterns recorded for the 35 nm copper films are presented in Figure 3. Diffraction peaks related to Cu are observed at 2θ = 43.3${}^{\circ }$ (111) and 50.5${}^{\circ }$ (200) [18]. Diffraction peaks for 2θ above 69${}^{\circ }$ (400) and 78${}^{\circ }$ (331) are associated with the Si substrate [19]. The average sizes of Cu crystallites in the 35 nm layers (<*D*>) are about 21 and 28 nm for an evaporation rate of 0.5 and 5.0 Å/s, respectively. The <*D*> values were calculated by the Scherrer formula [20]:

(6) $$<D>=\frac{0.9\lambda }{\beta \cos(2\theta )}.$$

where λ—the X-ray wavelength, and β—the full-width at half-maximum (FWHM) of the Bragg diffraction peak at angle 2θ.

**Figure 4:**
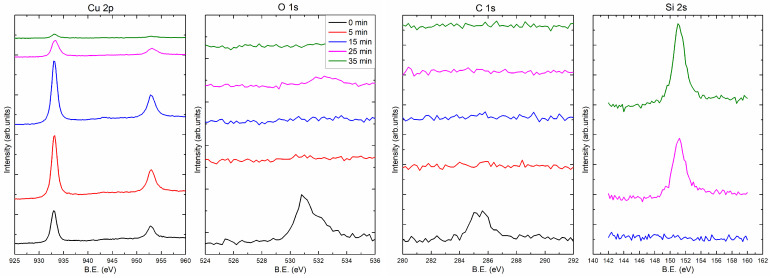
XPS spectra for 35 nm Cu layers prepared at the deposition rate 0.5 Å/s. The Si 2s spectra for 0 min and 5 min of ion sputtering were not recorded.

**Figure 5:**
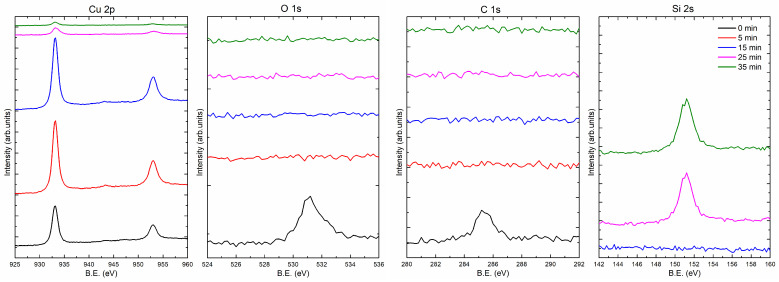
XPS spectra for thin Cu layers prepared at the deposition rate 5.0 Å/s. The Si 2s spectra for 0 min and 5 min of ion sputtering were not recorded.

**Figure 6:**
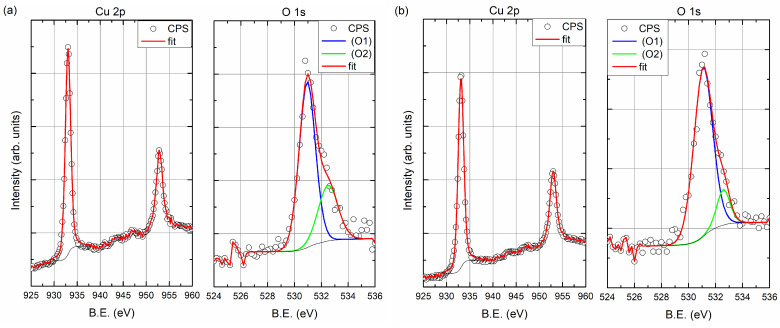
X-ray photoelectron spectroscopy (XPS) spectra (Cu 2p and O 1s peaks) of the sample deposited at room temperature (RT) with evaporation rate: (**a**) 0.5 Å/s and (**b**) 5.0 Å/s.

Figure 4 and Figure 5 present the XPS spectra showing the composition of the 35 nm copper layers obtained at the rates of 0.5 and 5.0 Å/s, respectively. Measurements were made at different depths of the film. For this purpose, the coating was sputtered with the ion beam (Ar${}^{+}$) for 0, 5, 15, 25, and 35 min. In part (a) of Figure 4 and Figure 5, there are spectral fragments containing two peaks derived from copper. These peaks are around 933.0 eV (2p${}_{3/2}$) and 952.8 eV (2p${}_{1/2}$). After 25 min of ion sputtering, the intensity of the Cu peaks decreased significantly, while a substrate peak (Si 2s) appeared (around 151 eV; see Figure 4d and Figure 5d), which indicates that the layers had been removed. Figure 4b,c show carbon and oxygen regions of the spectrum, respectively. The C 1s peak centered at 285 eV corresponds to carbon adsorbed from the atmosphere to the surface when the samples were removed from the vacuum chamber. As can be seen from the spectrum after 5 min of sputtering, there is no carbon left in the sample. The situation is similar for oxygen, after 5 min of sputtering with the ion beam, the oxygen is undetectable. The oxygen (O 1s) peak is in the region of 530–533 eV and has two components. Decomposition of O 1s peak is presented at Figure 6. The first one (O1, at 531 eV) is the most probably associated with CuO and the other one Cu${}_{2}$O (O2, at 532.5 eV) may be related to copper hydroxides formed at the surface [21]. For the layer produced with an evaporation rate of 0.5 Å/s, the oxygen peaks are visible after 25 min of ion sputtering. After this time, the Si peak is also visible; thus, the O 1s signal can be associated with the native oxide of the silicon substrate. It should be noted that the oxygen was not found in the inside of the copper film.

**Figure 7:**
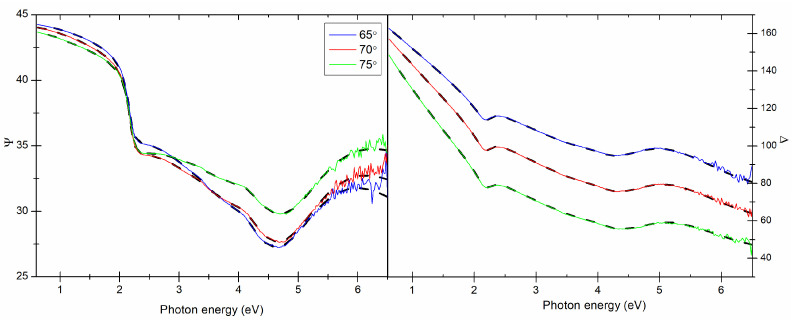
Ψ and Δ angles measured for three angles of incidence (65${}^{\circ }$, 70${}^{\circ }$, 75${}^{\circ }$) and their model fits for the Cu (35 nm, *v* = 5.0 Å/s), solid lines for experimental data, dashed lines are calculated spectra.

**Figure 8:**
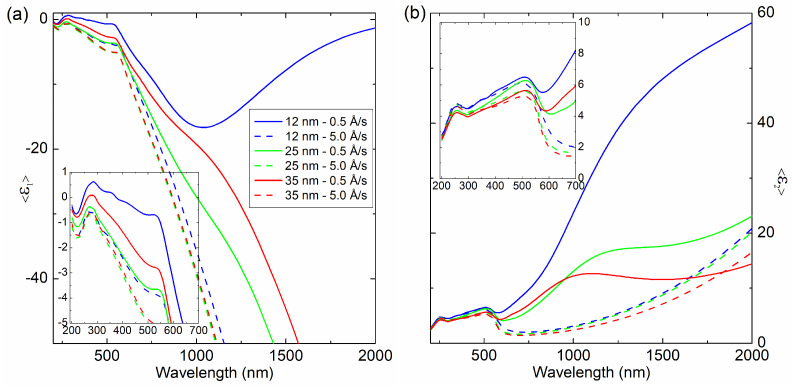
(**a**) Real (<$\varepsilon_{1}$>) and (**b**) imaginary (<$\varepsilon_{2}$>) parts of the effective dielectric function for the produced samples.

The results of spectroscopic ellipsometry (SE) measurements and the optical model of sample (substrate-Si∖SiO${}_{2}\backslash$Cu∖ambient, where SiO${}_{2}$ is the native oxide with a thickness of 2.5 nm) were used to determine the thickness ($d_{SE}$) and the effective complex dielectric function (<$\tilde{\varepsilon }$>) of the Cu films. The optical constants of Si and SiO${}_{2}$ were taken from the database of optical constants [22]. An example of the experimental and calculated ellipsometric data are presented in Figure 7. Based on the Ψ and Δ azimuths collected in the measurement, the real and imaginary parts of the dielectric function were determined (see Figure 8).

We use an effective dielectric function (<$\tilde{\varepsilon }$>), due to the fact that our coatings are not perfect and contain imperfections related to the roughness of the layer and the existence of voids between the grains. The effective dielectric function of the copper films was parameterized as in the following formula [22,23]:

(7) $$\begin{array}{c} <\tilde{\varepsilon }>=\varepsilon_{\infty }-\frac{{\left(\hbar \omega_{p}\right)}^{2}}{E^{2}-iE\left(\hbar \Gamma \right)}+\sum_{k}Lor.\left(A_{k},E_{k},\gamma_{k}\right)+LorPB\left(A,E_{top},\gamma ,E_{bot}\right). \end{array}$$

In Equation (7), $\varepsilon_{\infty }$ is the high-frequency dielectric constant, *E* is the energy of incident light, and $\hbar \omega_{p}$ and Γ represent the unscreened plasma energy and the free-carrier damping, respectively. $A_{k,j}$, $E_{k,j}$, and $\gamma_{k,j}$ are the amplitude, energy, and broadening of the *k*th and *j*th absorption band, respectively. The parabolic band model (LorPB) is an oscillator that converts narrowly broadened Lorentz oscillators with a parabolic JDOS (joint density of states) function [22]. This oscillator can be used to model the optical properties of noble metals (Au, Ag, and Cu) and their alloys [24]. The parameters of the effective dielectric function (<$\tilde{\varepsilon }$>) (Equation (7)) were varied to minimize the reduced mean squared error $\chi^{2}$ [22,23]:

(8) $$\chi^{2}=\frac{1}{M-P}\sum_{l}\left({\left(\frac{\Psi_{l}^{mod}-\Psi_{l}^{exp}}{\sigma_{\Psi_{l}}}\right)}^{2}+{\left(\frac{\Delta_{l}^{mod}-\Delta_{l}^{exp}}{\sigma_{\Delta_{l}}}\right)}^{2}\right),$$

where *M*—the total number of measured Ψ and Δ values, and *P*—the number of fitted model parameters. The quantities with the superscript mod and exp correspond to the calculated and measured ellipsometric azimuths, respectively. Quantities $\sigma_{\Psi_{l}}$ and $\sigma_{\Delta_{l}}$ represent the standard deviations of the experimental data. The $\chi^{2}$ value is less than 1.9 for all those fits.

**Figure 9:**
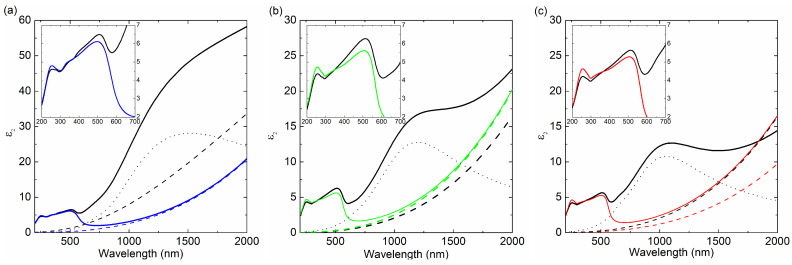
Decomposition of <$\varepsilon_{2}$> (solid lines: (**a**) Cu(12 nm), (**b**) Cu(25 nm), and (**c**) Cu(35 nm)) on the Drude term (dashed lines) for the Cu films deposited at *v* = 5.0 Å/s (color line) and *v* = 0.5 Å/s (black lines) and interband transitions (see inserts). The doted lines represent additional absorption band.

In the spectra of 200–600 nm, peaks related to electronic transitions are visible. At 600 nm, the band characteristic for copper is visible, corresponding to the $5d^{10}$–$6s^{1}$ interband transition [25]. In the 800–2000 nm region, a strong increase in <$\varepsilon_{2}$> can be observed. This feature is related to the Drude term, which is associated with interaction of the incident light with free carriers. This fact proves the metallic nature of the film (the film is electrically conductive). In this region, for layers obtained at a rate of 0.5 Å/s, a large peak related to the granular structure of the film is visible. As the coating thickness increases, this peak shifts in the spectrum towards shorter wavelengths. This effect is better seen in Figure 9, which shows the decomposition of <$\varepsilon_{2}$> components (mathematical functions described particular parts of the <$\varepsilon_{2}$> spectrum). As can be seen for the 12 nm layer, this peak is centered at 1500 nm and then shifted to about 1200 nm (for the d= 25 nm layer) and, finally, to about 1000 nm (for the 35 nm film). This shift shows that as the coating thickness increases, the grain size decreases. The change in film thickness clearly influences the grain size change, which affects on the effective dielectric function. As was mentioned earlier, the <$\tilde{\varepsilon }$> includes the Drude term. Its parameters (plasma energy—$\hbar \omega_{p}$; free-carrier damping—Γ) are summarized in Table 2. In general, the plasma energies for the layers made with v= 0.5 Å/s exhibit higher values than those for the films obtained at evaporation rate of 5.0 Å/s (taking into account the same thicknesses of the Cu layer). The lowest $\hbar \omega_{p}$ value of 6.92 eV was achieved for a coating with a thickness of 25 nm (v= 0.5 Å/s), whereas the lowest value of plasma energy was obtained for the layer 12 nm (0.5 Å/s) − 5.18 eV.

Based on the Drude parameters, we can calculate the mean relaxation time of conduction electrons (τ) and the optical resistivity ($\rho_{opt.}$). Their parameters are given by Equations [22,23]:

(9) $$\rho_{opt.}=\frac{\Gamma }{\varepsilon_{0}\omega_{p}^{2}},$$

and

(10) $$\tau =\Gamma^{-1},$$

where $\varepsilon_{0}$ is free-space permittivity. The τ value increases with increases in the thickness of the layer and the deposition rate. The lowest value of τ is ∼0.8 fs, and the highest is 11.6 fs for 12 nm (0.5 Å/s) and 35 nm (5.0 Å/s), respectively. The value of optical resistivity ($\rho_{opt.}$) decreases with the increase in the thickness and the evaporation rate. This parameter is the lowest for the 35 nm Cu film and is 6.05 and 10.9 μΩcm for the *v* equal 5.0 and 0.5 Å/s, respectively. The highest $\rho_{opt.}$ value of 226 μΩcm is for the 12 nm (0.5 Å/s) layer, but this result is subject to a relatively high mismatch error.

**Figure 10:**
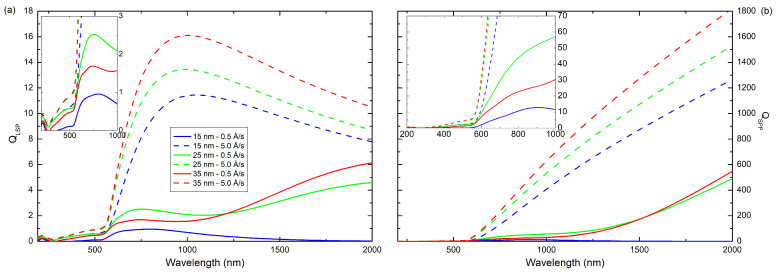
Comparison of calculated quality factor for (**a**) local surface plasmon and (**b**) surface plasmon polariton of produced samples.

Using Equations (4) and (5) and determined <$\varepsilon_{1}$> and <$\varepsilon_{2}$>, we calculated the quality factors (*Q*) for localized surface plasmon (LSP) and surface plasmon polaritons (SPP), which are shown in Figure 10. For layers prepared with *v* = 5.0 Å/s the maximum of $Q_{LSP}$, in the investigated wavelength range, is at about 1000 nm. The maximum $Q_{LSP}$ values are 11.4, 13.4, and 16.1 for coatings with thickness 12, 25, and 35 nm, respectively, whereas the maximum $Q_{SPP}$ is at 2000 nm and is 1265, 1531, and 1823 for films with thickness 12, 25, and 35 nm, respectively, while the films obtained at lower deposition rate have much lower quality factors. It means that the surface plasmons in these layers are more strongly suppressed. This may be because these films are more rougher. The $Q_{LSP}$ and $Q_{SPP}$ values for selected incident wavelengths are presented in Table 3. It is clearly visible that values of quality factors are several times (about one order of magnitude) lower for coatings produced with an evaporation rate of 0.5 Å/s than for layers produced with a rate of 5.0 Å/s.

Low values of the $R_{a}$ and $R_{q}$ parameters (see Table 1) and the layer’s thickness determined from ellipsometric measurements ($d_{SE}$; see Table 2), which are several dozen percentage points higher than the thickness determined from QCM (*d*), indicating the nanoporous structure of the films. The presence of nanopores in the coatings may also reduce the value of the quality factors $Q_{LSP}$ and $Q_{SPP}$. Additionally, the plasmonic effects can be attenuated by the oxidated surface of the films (oxygen was not detected inside the layer).

## 4. Conclusions

The investigated copper layers with thicknesses of 12, 25, and 35 nm were thermally deposited on silicon substrate under very low vacuum. In fact, despite the low vacuum during the deposition of the films, the films are not contaminated inside with oxygen (residual gases), as shown by XPS measurements. Oxygen exists only at the surface of the layer, and this is the result of oxidation and hydro-oxidation of the sample surface after they are removed from the vacuum chamber. More oxygen was adsorbed on coatings evaporated at a lower deposition rate (*v* = 0.5 Å/s). This is related to the more extended surface, as indicated by AFM images and roughness parameters. The dielectric functions of the Cu layers obtained at *v* = 0.5 Å/s show the existence of a strong absorption peak in them related to the granular structure of the film (the size effect), which shifts to shorter wavelengths with increasing coating thickness (this was to be expected). Electrons are more efficiently scattered in these layers, which causes the films to have a higher optical resistance than layers produced at higher evaporation rates. The scattering phenomenon also adversely affects the plasmonic properties of the coatings. The values of $Q_{LSP}$ and $Q_{SPP}$ parameters are much higher for the films fabricated with the deposition rate of 5.0 Å/s, so they are the ones that show better plasmonic properties. In this manuscript, we showed that the thickness of a layer and the deposition rate are crucial parameters which affect properties of the copper produced films. Moreover, we have explained the influence of growing conditions on microstructure and optical properties of the metallic layers and thus on their plasmonic properties.

## Figures and Tables

**Table 1:** The average roughness ($R_{q}$) and the root mean square roughness ($R_{a}$) parameters of the Cu thin films (for a scan size 1 μm × 1 μm) estimated using AFM.

Sample	$R_{q}$ (nm)	$R_{a}$ (nm)	$R_{\max}$ (nm)
Cu(35) *v* = 5.0 Å/s	0.93 ± 0.02	0.75 ± 0.02	7.6
Cu(35) *v* = 0.5 Å/s	1.37 ± 0.04	1.08 ± 0.03	12.7
Cu(25) *v* = 5.0 Å/s	0.71 ± 0.02	0.88 ± 0.01	6.6
Cu(25) *v* = 0.5 Å/s	1.35 ± 0.02	1.05 ± 0.02	14.4
Cu(12) *v* = 5.0 Å/s	0.40 ± 0.10	0.30 ± 0.10	6.1
Cu(12) *v* = 0.5 Å/s	0.90 ± 0.10	0.67 ± 0.05	13.3

**Table 2:** The thickness of Cu layer ($d_{SE}$) determined from spectroscopic ellipsometry measurements, the plasma energy ($\hbar \omega_{p}$), the free-carrier damping (ℏΓ), the mean relaxation time of conduction electrons (τ) and the optical resistivity ($\rho_{opt.}$).

Sample	$d_{\mathrm{SE}}$ (nm)	$\hbar \omega_{p}$ (eV)	ℏΓ (eV)	τ (fs)	$\rho_{\mathrm{opt}.}$ (μΩcm)
Cu(35) *v* = 5.0 Å/s	49.9 ± 0.2	8.34 ± 0.01	0.0567 ± 0.0009	11.60 ± 0.20	6.1 ± 0.1
Cu(35) *v* = 0.5 Å/s	47.3 ± 0.1	6.32 ± 0.02	0.0586 ± 0.0024	11.20 ± 0.50	10.9 ± 0.5
Cu(25) *v* = 5.0 Å/s	31.9 ± 0.2	8.42 ± 0.01	0.0684 ± 0.0010	9.63 ± 0.15	7.2 ± 0.1
Cu(25) *v* = 0.5 Å/s	37.3 ± 0.5	6.92 ± 0.02	0.0835 ± 0.0016	7.88 ± 0.15	13.0 ± 0.3
Cu(12) *v* = 5.0 Å/s	15.1 ± 0.1	8.13 ± 0.02	0.0758 ± 0.0007	8.68 ± 0.08	8.5 ± 0.1
Cu(12) *v* = 0.5 Å/s	18.1 ± 0.1	5.18 ± 0.28	0.8160 ± 0.0970	0.81 ± 0.10	226 ± 36

**Table 3:** The values of the quality factor for LSP and SPP at certain incident wavelengths 650, 1000, and 1550 nm.

Sample	$Q_{\mathrm{LSP}}$			$Q_{\mathrm{SPP}}$		
	**650 nm**	**1000 nm**	**1550 nm**	**650 nm**	**1000 nm**	**1550 nm**
Cu(35) *v* = 5.0 Å/s	12.6	16.1	13.0	231.7	627.6	1330.9
Cu(35) *v* = 0.5 Å/s	1.7	1.6	4.2	19.6	30.3	202.1
Cu(25) *v* = 5.0 Å/s	10.8	13.4	10.8	200.1	531.9	1119.8
Cu(25) *v* = 0.5 Å/s	2.5	2.1	3.3	37.2	57.2	199.4
Cu(12) *v* = 5.0 Å/s	8.1	11.4	9.6	129.5	407.5	915.0
Cu(12) *v* = 0.5 Å/s	0.9	0.7	0.1	8.4	11.5	0.9

## Data Availability

Effective dielectric functions of Cu thin layers are available (since 15 December 2021): https://doi.org/10.18150/EXKMPS.
